# Chloride Treatments Improve Zinc Telluride Absorbers for Photoelectrochemical Carbon Dioxide Reduction

**DOI:** 10.1021/acsaem.4c02498

**Published:** 2025-01-07

**Authors:** Christopher P. Muzzillo, Yungchieh Lai, Joel A. Haber, Andriy Zakutayev

**Affiliations:** † National Renewable Energy Laboratory, Golden, Colorado 80401, United States; ‡ California Institute of Technology, Pasadena, California 91125, United States

**Keywords:** ZnTe, CO_2_, PEC, PEC CO_2_ RR, MnCl_2_, MgCl_2_

## Abstract

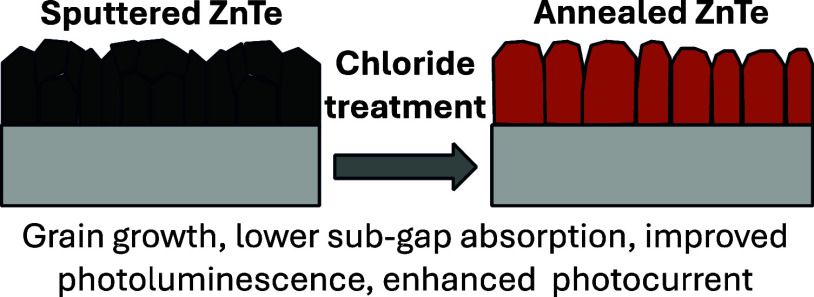

Utilizing sunlight for photoelectrochemical carbon dioxide reduction reaction (PEC CO_2_ RR) is a carbon-neutral path to valuable liquid fuels. Higher quality photoabsorbers are needed to improve the efficiency of the PEC CO_2_ RR process. We show how the optoelectronic properties of sputtered ZnTe absorbers can be improved for this purpose via chloride treatments. MnCl_2_ and MgCl_2_ heat treatments recrystallize ZnTe absorbers to enlarge grains and improve photoluminescence. These material improvements result in the highest PEC CO_2_ RR photocurrent density reported for planar ZnTe and >50% Faradaic efficiency to CO formation with diaryliodonium additive in the solution. These results pave the way to integration of polycrystalline thin-film photoabsorbers in PEC CO_2_ RR systems.

## Introduction

Long-haul aviation and other demanding sectors of the economy require hydrocarbon fuel,^1,2^ which makes completely eliminating carbon-based liquid fuels difficult. Instead, CO_2_ captured from the atmosphere can be sourced to create carbon-neutral fuel. However, converting CO_2_ into usable products is energy intensive, with every step requiring more energy input. To potentially solve all of these issues, the prospect of harvesting sunlight to directly drive the photoelectrochemical carbon dioxide reduction reaction (PEC CO_2_ RR) is technologically interesting.^3^

There are many photoabsorber materials that have been experimentally studied for PEC CO_2_ RR applications: photovoltaic (PV)-grade silicon (Si), III–V semiconductors (GaP, GaN, GaAs, and InP), II–VI semiconductors (CdS, CdTe, CdSe, ZnTe, and ZnSe), Cu-based chalcopyrites and kesterites (CuInS_2_, CuInSe_2_, Cu(In,Ga)Se_2_, and Cu_2_ZnSnS_4_), and other chalcogenides (Bi_2_S_3_, MnS, WSe_2_, MoSe_2_, and MoS_2_), as well as various metal oxides (Bi_2_WO_6_, BaTiO_3_, BiVO_4_, CeO_2_, CuGaO_2_, etc.) and carbides (SiC and C_3_N_4_).^3,4^ Still more CO_2_ RR photocathodes have been proposed by theoretical calculations based on the combination of their PEC stability and optoelectronic properties.^4^

**Figure 1:**
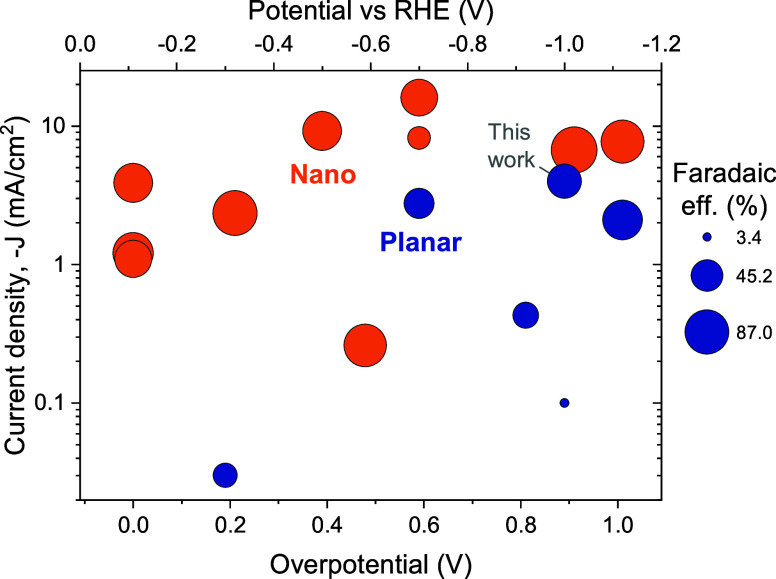
Literature results for ZnTe PEC CO_2_ RR: current density as a function of CO_2_ RR overpotential (bottom) or potential vs RHE (top) for planar ZnTe devices (blue) or nanostructured ZnTe (orange), with circle area proportional to Faradaic efficiency, showing that this work has the highest photocurrent density for planar ZnTe. Values and references are in Table S2.

Among many candidates, ZnTe’s bands straddle the CO_2_ reduction and water oxidation potentials.^4,5^ ZnTe also has favorable stability, optical absorption, charge transport properties, and low overpotential, making it an attractive photoabsorber for PEC CO_2_ RR.^5−15^ ZnTe is more commonly used as a hole transport layer in CdTe PV,^16−19^ although it has also been used as a photoabsorber.^20−22^ Most ZnTe CO_2_ RR research uses nanostructured surfaces, a well-established route to enhancing PEC current density to over 10 mA/cm^2^ at −0.5 V vs reversible hydrogen electrode (RHE) with Faradaic efficiency of >50% (Figure 1).^5−13,15^ Eliminating nonradiative recombination in bulk ZnTe films with planar surfaces grown with scalable methods is an alternative approach to improve ZnTe for PEC CO_2_ RR, but it is showing smaller photocurrent and lower Faradaic efficiency so far.^8,12−15^ Such planar samples help separate surface morphology effects from the charge carrier properties of the ZnTe absorber and can eventually be coupled with nanostructuring to further enhance performance.

Organic additives have been widely investigated to enhance the performance of CO_2_ RR both on dark electrodes^23−26^ and during PEC at semiconductor surfaces.^27,28^ While many investigations with organic coatings were derived from N-arylpyridinium,^29^ diaryliodonium and similar salts were recently reported to enhance CO_2_ RR not only for selectivity compared to hydrogen evolution reaction (HER) but also activity. Furthermore, they were reported to be robust on the electrode even at higher electrode surface temperatures.^30^ These advantages along with its easy accessibility (commercial availability) make diaryliodonium desirable for enhancing the performance of photocathodes for CO_2_ RR.

In this work, we report novel chloride treatment conditions that improve the structural and optoelectronic quality of ZnTe thin films and demonstrate their PEC CO_2_ RR applications. In particular, annealing sputtered ZnTe thin films in a MnCl_2_ atmosphere induces recrystallization, enlarging grains, and increasing photoluminescence (PL) intensity. These chloride treatments enhance PEC CO_2_ RR to the measured photocurrent density that would extrapolate to −4.0 mA/cm^2^ at −1.0 V vs RHE—among the highest for planar ZnTe thin films. Molecular diaryliodonium salts as additives to the solution enhance the measured Faradaic efficiency for CO formation up to 50.4%, showing the promise of this chloride-treated ZnTe for CO_2_ RR applications.

## Methods

**Figure 2:**
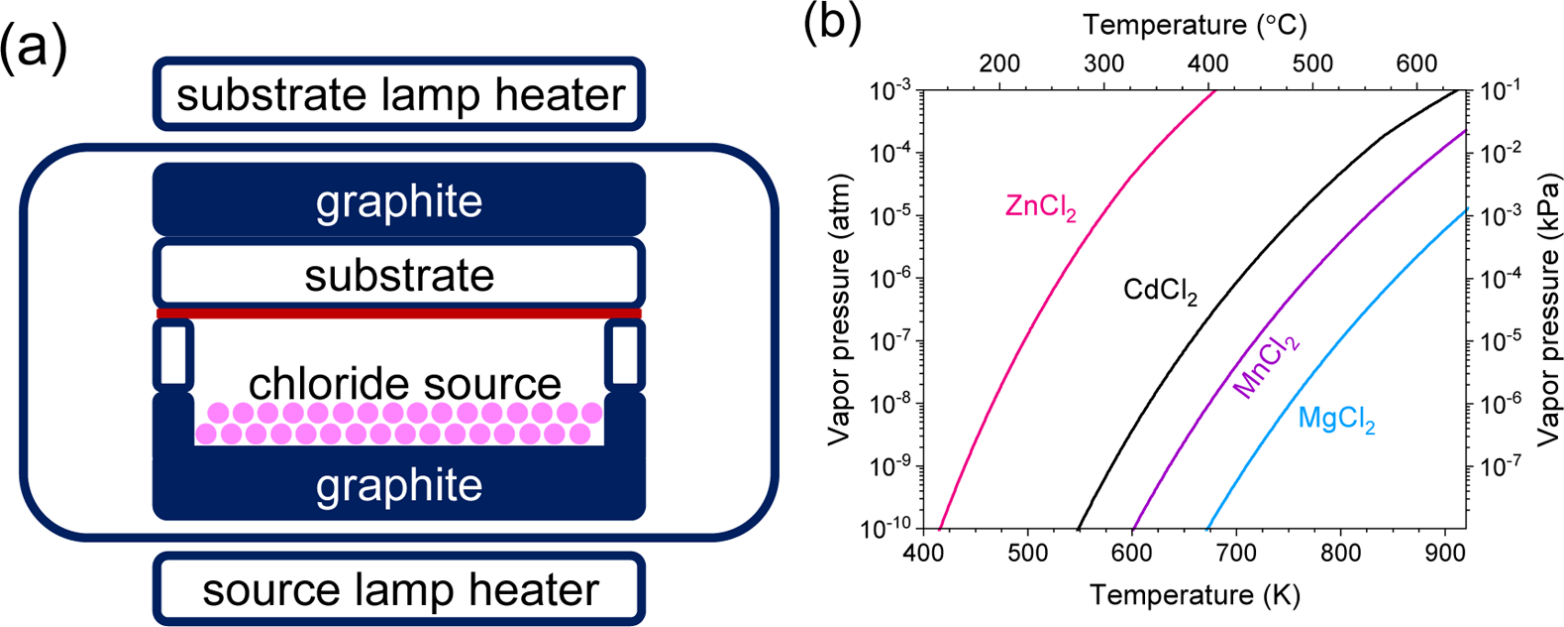
(a) Process schematic for the chloride treatment of ZnTe films and (b) vapor pressure (left axis atm; right axis kPa) as a function of temperature (bottom axis K; top axis °C) for ZnCl_2_ (red), CdCl_2_ (black), MnCl_2_ (purple), and MgCl_2_ (blue), showing that MnCl_2_ and MgCl_2_ have much lower relative volatility.

We radio frequency (RF) sputter ZnTe films (∼1 μm) at 50 W onto soda–lime glass/SnO_2_/SiO_2_/F:SnO_2_ (Tec-15) substrates held at 170 °C over the course of 70 min. We load manganese chloride tetrahydrate (MnCl_2_·4H_2_O) or magnesium chloride (air-exposed MgCl_2_ is likely present as MgCl_2_·6H_2_O^31^) into a graphite boat. We place the ZnTe films above the crystals with a source–substrate separation of 3 mm. The graphite boat and lid have holes for independent thermocouples (Figure 2). In a cold-wall quartz reactor filled with 400 Torr inert He gas, we heat the source to 400–550 °C while heating the substrate to 35 °C cooler than the source and holding for 30 min. We prebake the source and substrate to 200 °C and hold for 10 min before the chloride treatments to desorb moisture (unless noted). We perform thermochemical equilibrium calculations with Thermo-Calc and the Scientific Group Thermodata Europe 1994 substance database.

We perform symmetric θ–2θ X-ray diffraction (XRD) with monochromated Cu Kα radiation. We measure film composition by X-ray fluorescence (XRF) for 60 s at 10 locations spread across each 1 in. ×1 in sample. We perform ultraviolet–visible (UV/visible) spectroscopy using a Cary 6000 spectrophotometer with a diffuse reflectance-integrating sphere. We combine transmissivity, reflectivity, and thickness data to construct Tauc plots.^32^ After squaring the product of absorptivity (α), Planck’s constant (h), and photon frequency (ν), we use a least-squares fit on the linear region and extrapolate to α = 0 to calculate direct band gap. We collect room temperature spectrally resolved PL on a Raman microscope using a 50× lens with 0.35 numerical aperture and 532 nm laser excitation at 50 μW with a spot size of 40 μm × 65 μm (5.1 × 10^18^ photons/cm^2^ s). Using lower fluence for PL does not change the trends we report here. We perform room temperature time-resolved PL (TRPL) by exciting with a 405 nm laser at low fluence (4.2 × 10^16^ photons/cm^2^ s, where 1 sun is 2 × 10^17^ photons/cm^2^ s). We use either a 550 nm bandpass filter with a 40 nm full width at half-maximum or a 700 nm long-pass filter at the detector to study band edge TRPL and defect emission TRPL, respectively. We fit TRPL data to biexponential functions to extract short and long minority carrier lifetimes (τ_1_ and τ_2_, respectively).

We use 0.1 M KHCO_3_ electrolyte, with or without 10 mM diaryliodonium additive,^33^ 455 nm light-emitting diode (LED) illumination at 11 mW/cm^2^, and 10 mV/s sweeps from +0.2 to −1.0 V vs RHE for PEC chopped-light cyclic voltammetry (CV) and for chronoamperometry (CA). We select a 455 nm LED (Thorlabs M455F3) because it has higher energy than the band gap of ZnTe and is in the range of visible light, not ultraviolet. We perform multipotential measurements (by CA) following CV to investigate the CO_2_ RR product distribution. We carry out CA measurements in the order 0, −0.2, −0.4, −0.6, −0.8, and −1 V vs RHE for 15 min unless the film mechanically delaminates. The high-throughput analytical electrochemistry (HT-ANEC) instrument^34^ we use to test ZnTe CO_2_ RR performance requires rapid flow to generate suitable and reproducible mass transport conditions. The rapid flow could cause the predeposited additive film to delaminate over time. For tests with additive, the electrolyte contains the additive at all times to ensure that the film remains on the electrode; hence, the thickness of diaryliodonium is not directly controlled. For this initial study, we focus on the discovery of the combined improvement in selectivity and activity. The impact of diaryliodonium thickness on stability and selectivity optimization will be the subject of future work. At the end of each (photo)electrolysis, we sample gaseous and liquid products by the robotic sample handling system and analyze by gas chromatography (GC; Thermo Scientific TRACE 1300) and high-performance liquid chromatography (Thermo Scientific UltiMate 3000), respectively. Detailed product detection methods are in a previous publication.^34^ Stability due to (photo)corrosion is known to occur in photocathodes for CO_2_ RR. We aliquot all pre- and post-PEC electrolytes at each potential to monitor photocathode dissolution. We use inductively coupled plasma mass spectrometry (ICP–MS) by a Thermo Fisher Scientific iCAP RQ instrument to determine the concentration of dissolved metals in electrolytes used for (photo)electrochemistry.

### Thermodynamic Calculation Results

The CdCl_2_ treatment is a well-established method to improve the quality of Cd(Se,Te) polycrystalline thin-film PV absorbers. CdCl_2_ (or MgCl_2_^35^) recrystallizes Cd(Se,Te) to increase grain size and passivate by reducing nonradiative recombination of photogenerated minority charge carriers.^36^ However, Cd(Se,Te)’s successful chloride treatment cannot be used directly on ZnTe for two reasons: (1) ZnCl_2_’s vapor pressure is more than 3 orders of magnitude higher than CdCl_2_ (Table 1 and Figure 1), so it is too volatile for high-temperature chloride treatments at 400–450 °C. (2) CdCl_2_ cannot be used to treat ZnTe because the undesired formation of CdTe and volatile ZnCl_2_ is thermodynamically favorable (Table 2).^37−39^ A previous effort employed a barrier or capping layer to trap ZnCl_2_ within ZnTe films, reducing ZnCl_2_ vapor loss,^39^ but for PEC applications, such a barrier layer would constrain device architecture and obscure changes to the ZnTe absorber itself. Another work used independent CdCl_2_, ZnCl_2_, and/or MgCl_2_ reactant sources to prevent Zn loss in (Cd,Zn)Te alloys.^40^ Former studies annealed (Cd,Zn)Te,^41^ treated (Cd,Mn)Te with CdCl_2_,^42^ and prevented Mn loss from (Cd,Mn)Te alloys by using a MnCl_2_ + CdCl_2_ source,^43^ whereas here we target the wider band gap of ZnTe with no Mn alloying.

**Table 1:** Vapor Pressure of ZnC_2_, CdCl_2_, MnCl_2_, and MgCl_2_ Reactants, Showing that MnCl_2_ and MgCl_2_ Avoid the Volatility Issue That Prevents ZnCl_2_ Treatment of ZnTe

reactant	*P*_vap,725 K_ (·10^–3^ atm)
ZnCl_2_	4.2
CdCl_2_	2.6 × 10^–3^
MnCl_2_	1.4 × 10^–4^
MgCl_2_	2.4 × 10^–6^

**Table 2:** Gibbs Energy Change for CdCl_2_, MnCl_2_, or MgCl_2_ Reacting with ZnTe, Showing that MnCl_2_ and MgCl_2_ Avoid Undesired Products Such as Volatile ZnCl_2_

reaction	Δ*G*_rxn,725 K_ (kJ/mol)
ZnTe + CdCl_2_ → ZnCl_2_ + CdTe	–4.0
ZnTe + MnCl_2_ → ZnCl_2_ + MnTe	+21.7
ZnTe + MgCl_2_ → ZnCl_2_ + MgTe	+29.6

We propose that MnCl_2_ and MgCl_2_ can solve these issues with ZnTe chloride treatments because they are less volatile. Table 1 and Figure 1 show that MnCl_2_’s vapor pressure is 19x lower than CdCl_2_ at temperatures useful for a chloride treatment (725 K). Moreover, reacting ZnTe with MnCl_2_ to form the undesired MnTe and volatile ZnCl_2_ is thermodynamically unfavorable (Table 2). MgCl_2_ has even lower vapor pressure and less favorable Gibbs energy change for the undesired MgTe + ZnCl_2_ formation reaction. Mn and Mg both occur in the 2+ oxidation state like Zn, while MnTe and MgTe have wider band gaps (3.25 and 3.40 eV, respectively) than ZnTe, so Mn and Mg should be electronically benign in ZnTe films relative to other chloride sources. Therefore, both MnCl_2_ and MgCl_2_ are attractive for ZnTe chloride treatments because they have diminished volatility and reactivity.

### Annealing Results

**Figure 3:**
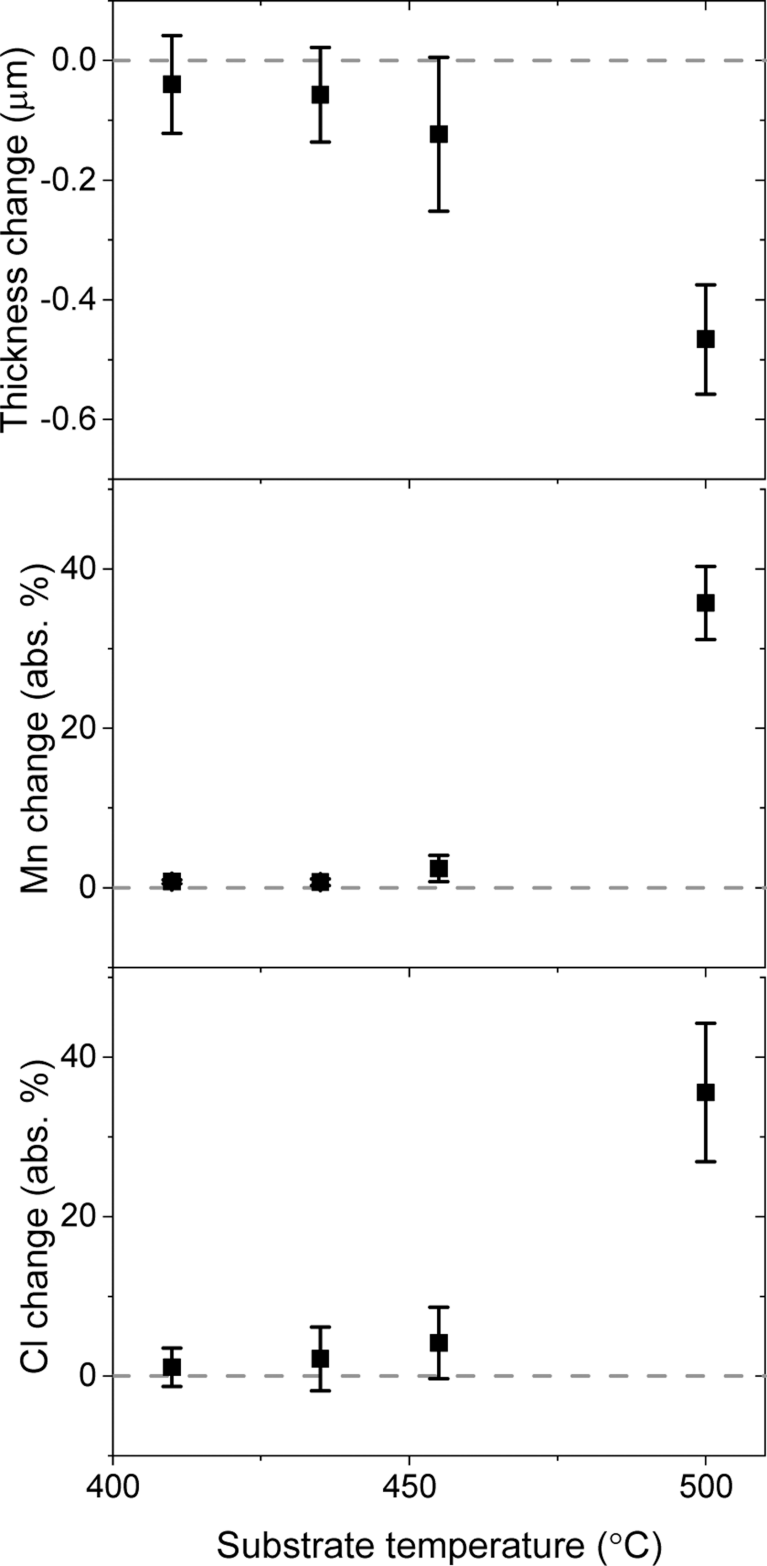
XRF data showing the change in ZnTe film thickness, Mn, and Cl composition after MnCl_2_ treatments as a function of substrate temperature, showing substrate temperatures above 435 °C etch the ZnTe.

We find that MnCl_2_ treatments above 475 °C and MgCl_2_ treatments above 425 °C etch the ZnTe film (435 and 390 °C substrate temperatures, respectively, see Figure 3), so we choose those source temperatures for further investigation. We perform a thermal anneal control using an empty graphite boat. The anneals do not decrease ZnTe film thickness by XRF, but they do slightly decrease Te content (Figure S1).^44^ On the other hand, the MnCl_2_ and MgCl_2_ treatments decrease film thickness and cause more significant Zn loss, indicating that etching occurs via ZnCl_2_ formation (Figure S1) similar to previous reports.^39,40^ When we leave MgCl_2_ in air instead of a N_2_-flow drybox and skip the prebake step to allow more H_2_O into the system, more etching occurs (Figure S2). The 200 °C prebake desorbs all 6 H_2_O molecules from MgCl_2_·6H_2_O^31^ and only 3 H_2_O molecules from MnCl_2_·4H_2_O,^45^ so the effect is small for the latter (Figure S2). HCl is expected to form from the sources,^31^ making hydrochloric acid in the presence of moisture to facilitate etching. We also find that repeated thermal cycling of the MnCl_2_ and MgCl_2_ sources reduces the resulting films’ PL peak intensity (Figure S3), indicating that the sources’ reactivity eventually diminishes, probably due to chlorine loss via HCl formation.

**Figure 4:**
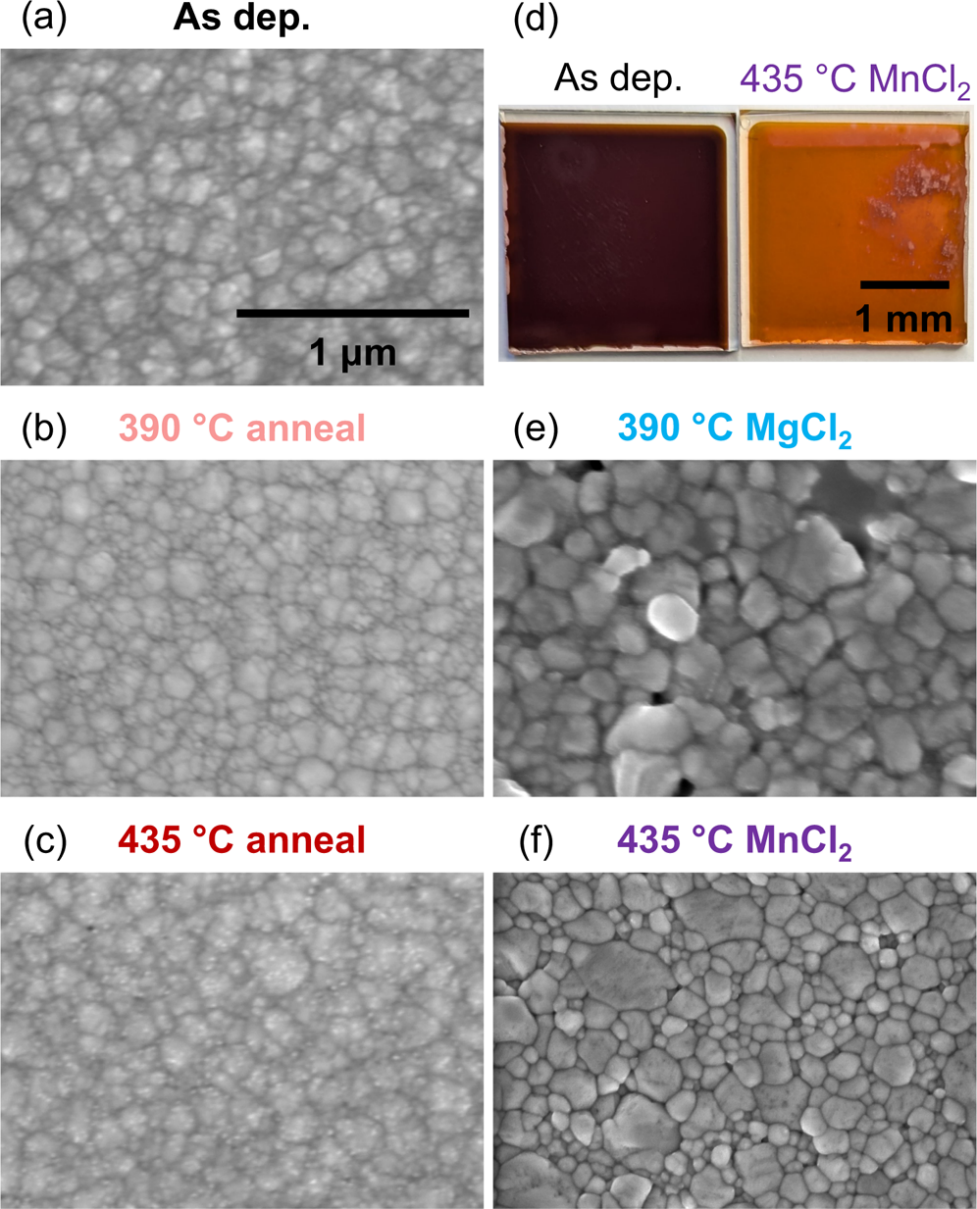
Scanning electron micrographs showing the (a) as-deposited, (b) 390 °C anneal, (c) 435 °C anneal, (e) 390 °C MgCl_2_, and (f) 435 °C MnCl_2_ samples in plan view, showing that the chloride treatments increase grain size and form smoother surfaces, relative to the anneals. The scale bar in (a) applies to (b,c,e,f). (d) Photographs of as-deposited (left) and 435 °C MnCl_2_ (right) samples.

**Figure 5:**
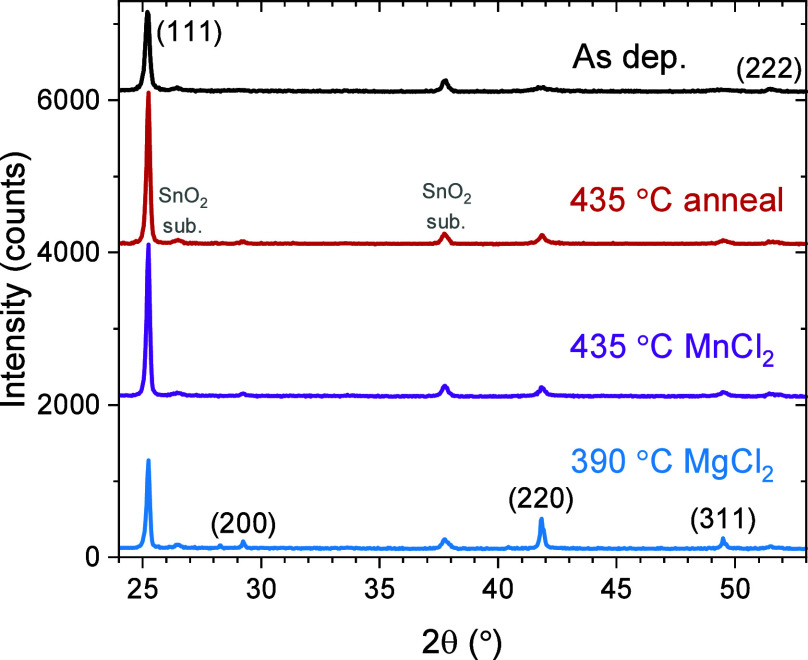
XRD for the as-deposited (black), 435 °C anneal (red), 435 °C MnCl_2_ (purple), and 390 °C MgCl_2_ (blue) samples, showing that annealing enhances crystallinity and the chloride treatments additionally reduce (111) texture, indicating recrystallization.

We compare grain size and morphology for the optimized chloride treatments in Figure 4. The as-deposited films have small grains 50–200 nm in diameter. Since the deposition is performed at 170 °C and is not far from the annealing temperature, the thermal anneals without chloride do not increase apparent grain size much. Relative to the thermal anneals, the chloride treatments increase grain size up to 500 nm and form smoother surfaces. Smoother surfaces and more rounded grains result from chloride recrystallization, as shown in Figure 4.^38,46^ According to XRD in Figure 5, as-deposited ZnTe thin films are polycrystalline in nature, with a (111) preferential orientation^44^ compared to the randomly oriented XRD reference pattern from ICSD. Annealing and chloride treatments enhance crystallinity by XRD, as shown in Figures 5 and S4. The chloride treatments additionally have reduced (111) texture closer to the random orientation in Figure 5, indicating recrystallization.

**Figure 6:**
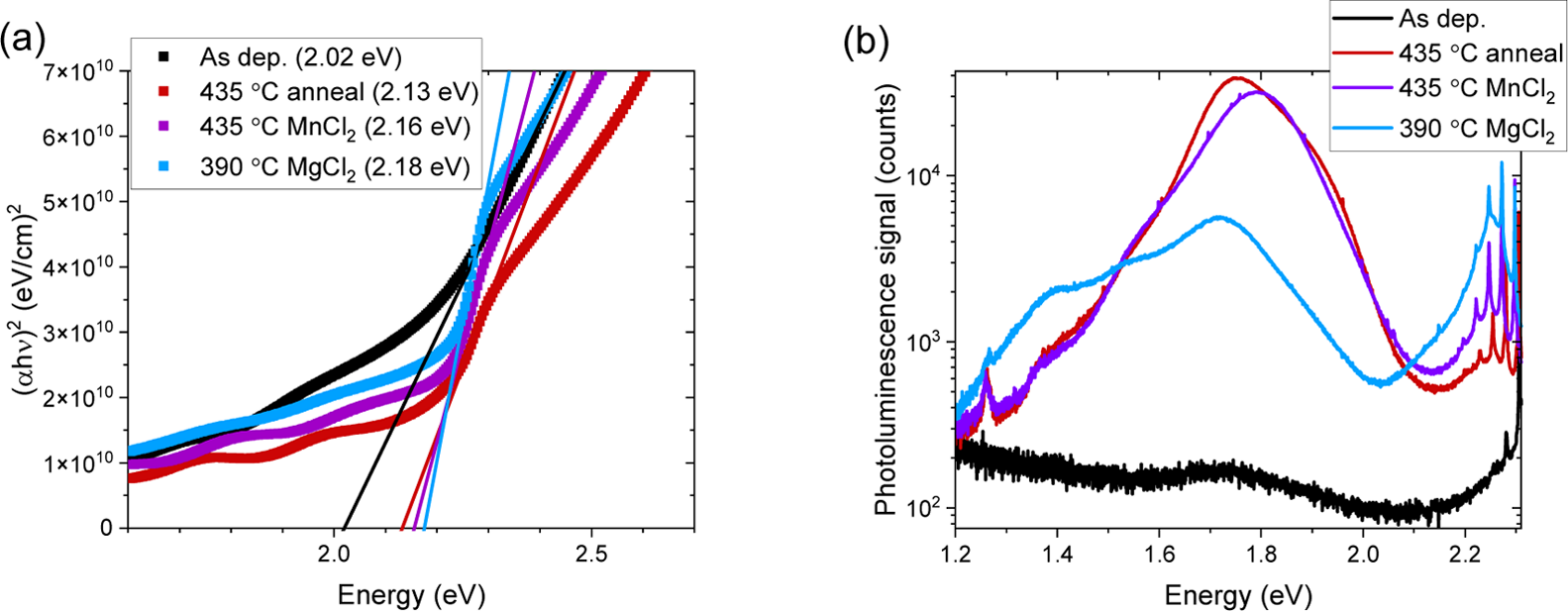
(a) UV/visible spectroscopy for the as-deposited (black; 2.02 eV), 435 °C anneal (red; 2.13 eV), 435 °C MnCl_2_ (purple; 2.16 eV), and 390 °C MgCl_2_ (blue; 2.18 eV) samples with fits to their linear regions extrapolated to give band gaps, showing that annealing sharpens the absorption onset and the chloride treatments sharpen it even more. (b) PL for the as-deposited (black), 435 °C anneal (red), 435 °C MnCl_2_ (purple), and 390 °C MgCl_2_ (blue) samples, showing that annealing enhances the defect PL emission at 1.75 eV, while chloride treatments enhance band edge PL emission at 2.25 eV, relative to the anneals.

As-deposited ZnTe films exhibit a finite signal absorption near the 2.2 eV band edge^18^ and almost no PL response (Figure 6). Annealing sharpens the absorption onset by UV/visible spectroscopy in Figures S5 and 6, and the chloride treatments sharpen the absorption onset even more, indicating enhanced crystallinity. In addition, annealing enhances the defect PL emission at 1.75 eV (Figure 6). That energy may be consistent with an oxygen-related localized defect.^20,47−50^ The chloride treatments enhance band edge PL emission at 2.25 eV, relative to the anneals (Figure 6). The anneals and chloride treatments reduce band edge TRPL lifetime but enhance defect emission lifetime (Figure S6 and Table S1). More study is needed to put minority carrier lifetimes into context with data on carrier concentration and mobility. For instance, improved band edge PL could coincide with trap elimination, which would decrease apparent band edge lifetime if it were dominated by detrapping.^51^ In summary, the optimized MnCl_2_ and MgCl_2_ treatments recrystallize ZnTe by balancing the formation of ZnCl_2_ with its evaporative loss, enhancing grain size, absorption onset, and band edge PL emission.

### Photoelectrochemical Results

**Figure 7:**
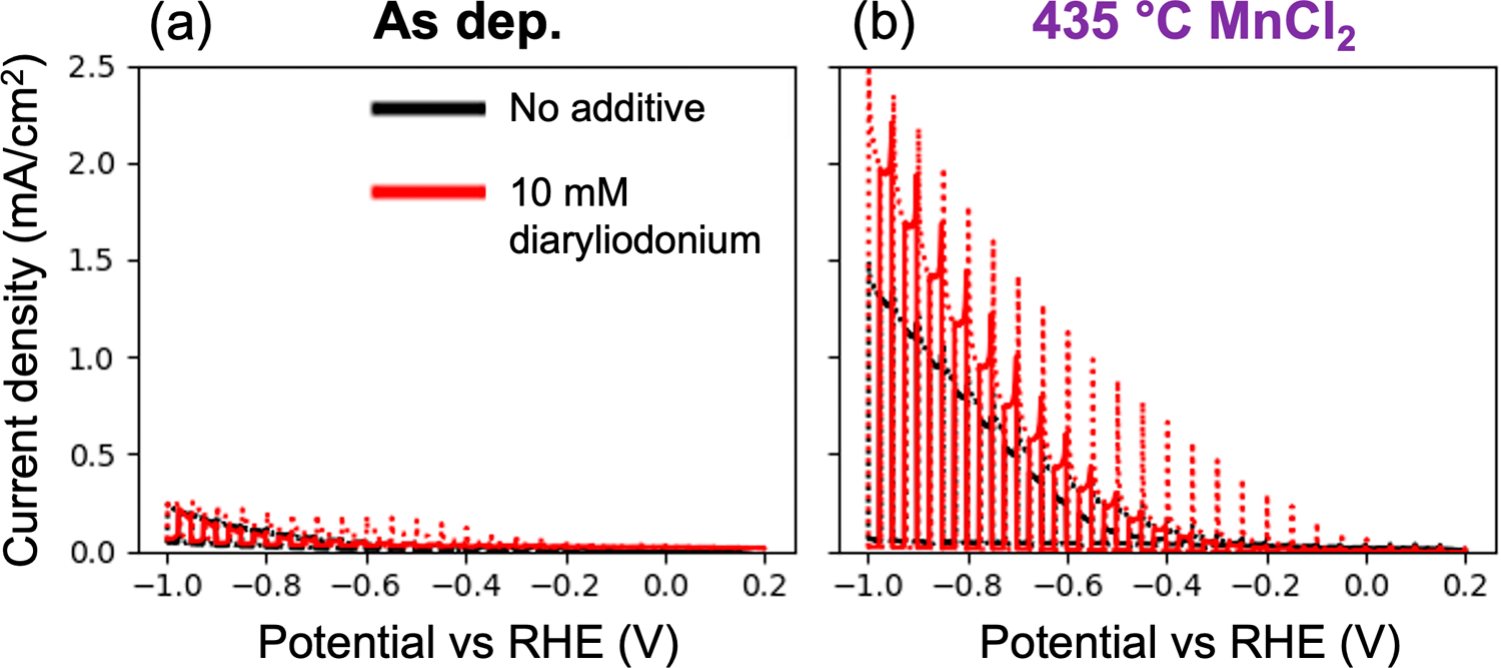
PEC current density–voltage data (3rd cycle) without (black lines) and with 10 mM diaryliodonium additive (red lines) for the as-deposited (a) and 435 °C MnCl_2_ (b) samples, respectively. The solid and dotted lines are cathodic and anodic sweeps, respectively, showing that the MnCl_2_ sample with additive has improved photocurrent density.

To evaluate the performance of the four ZnTe photocathodes investigated in this study, we perform 3 CV cycles followed by multiple CA measurements using the HT-ANEC instrument. We previously described this HT-ANEC instrument with fiber-optic-coupled photodiodes for front side illumination and demonstrated it to be an efficient instrument for PEC CO_2_ RR photocathode screening.^28^ The CV scans reveal that the annealed and MnCl_2_-treated absorbers have similar low dark currents and much higher photocurrents (1.0–2.1 mA/cm^2^ at −1.0 V vs RHE) compared to as-deposited samples (0.2 mA/cm^2^ at −1.0 V vs RHE) (Figures 7 and S7). The enhanced photocurrent correlates with the more intense band edge PL (Figure 6b) and the associated reduction in nonradiative recombination. We also perform chopped light CV scans on bare F:SnO_2_ substrates, which have dark currents at potentials more negative than −0.7 V vs RHE but no photocurrents in the potential range tested (Figure S7). The enhanced dark current in the MgCl_2_-treated ZnTe samples (Figure S7) probably stems from the underlaying substrate. While the MgCl_2_ treatment enhanced PL the most, its morphology suffered, and we attribute its inferior photocurrent to pinholes (Figure 4e) and delamination (Figure S8). Additionally, all samples have a reductive current between −0.25 and −0.6 V vs RHE, but present only in the first cycle of the cathodic sweep. The cause of these reductive currents could be the reduction of residues on the surface of ZnTe (e.g., TeO_*x*_ or ZnO).

The observation of a negligible dark current in these CV experiments down to −1.0 V vs RHE informed the choice of subsequent operating potentials between −0.2 and −1.0 V vs RHE such that the only current measured under illumination can be assumed to be the photocurrent. The higher photocurrents from the annealed and chloride-treated absorbers in the CA measurements agrees with those seen in the above CV scans. We note that the very high current at −1.0 V vs RHE seen in the as-deposited sample is from the (dark) current of the F:SnO_2_ substrate since its ZnTe film was totally delaminated after multipotential CA tests (Figure S8). Carbon monoxide (CO) is the only CO_2_ RR product and is minor, while H_2_ is the major product over all four photocathodes and at all potentials tested. In the low-bias regions, CO might be below the GC detection limit and hence not accounted for in Faradaic efficiency calculations (although there is likely no CO formation at these potentials). The higher applied bias regions have CO Faradaic efficiency of approximately 10%. We collect ICP–MS on the post-PEC electrolyte at each potential to evaluate if any dissolution of ZnTe occurs during the PEC tests (Figure S10). Compared to the pre-PEC concentrations (star symbols), the post-PEC electrolytes show that all sample types of ZnTe are stable for most conditions. The very high Zn concentration seen (mainly) for high bias regions is more likely due to (slight) film delamination (Figure S8), and it occurs in only a few samples.

Most additives investigated to enhance performance of CO_2_ RR^23−26^ have been shown to reduce and dimerize/oligomerize into a nonconductive layer on the electrode surface to enhance CO_2_ RR product selectivity by suppressing HER, which leads to reduced total photocurrent.^52^ Mechanisms proposed to account for the change in selectivity include slow diffusion of proton carriers to the electrode, lower H_2_O and increased CO_2_ concentration within the films, nanostructuring of the electrode, and interactions of CO_2_ reduction intermediates on the electrode with the film.^26,53^ More specific to the diaryliodonium additive used in this study, it was recently reported that beyond just suppressing HER, it could also enhance CO_2_ RR both in selectivity and activity on polycrystalline Cu electrodes.^30^ In addition to the above proposed mechanisms for suppression of HER, this grafted film has been proposed to grow perpendicular to the surface and create a low-density film with channels that may enable facile transport through the film, thereby increasing CO_2_ catalytic activity and maintaining overall current density.^30,54^

We thus select 10 mM diaryliodonium for studying the four ZnTe samples. The first cycle of CV scans (Figure S7) shows reductive currents at very positive potentials. It could result from polymerization/deposition of additives onto the ZnTe surface since they are not seen in the following cycles. However, the initial reductive current between −0.25 and −0.6 V versus RHE observed with no additive is suppressed when the additive is present. The additive may prevent or slow any sublayer metal or metal oxide from corroding.^28^ Overall, the additive does not change HER or CO_2_ RR onset potential. However, the additive results in comparable or slightly decreased photocurrents for as-deposited, annealed, and MgCl_2_-treated ZnTe samples (Figures 7 and S6), and it agrees with the CA data we discuss later. However, the MnCl_2_-treated ZnTe sample is an exception, with its photocurrents increasing slightly in the presence of additives. This is probably due to sample nonuniformity. PL measurements on several sample locations show that the chloride treatments introduce nonuniformity in optoelectronic quality (Figure S11). In other words, the MnCl_2_-treated sample area tested with the additive happened to have superior optoelectronic quality relative to the area tested with no additive. More uniform source material may eliminate varied photocurrents in the future. Overall, our observed currents with and without diaryliodonium over the four ZnTe photocathodes are comparable and in agreement with what was reported for this additive on CO_2_ RR.^30^ The high photocurrent density of up to −1.5 mA/cm^2^ at −1.0 V vs RHE was achieved for the MnCl_2_-treated sample.

**Figure 8:**
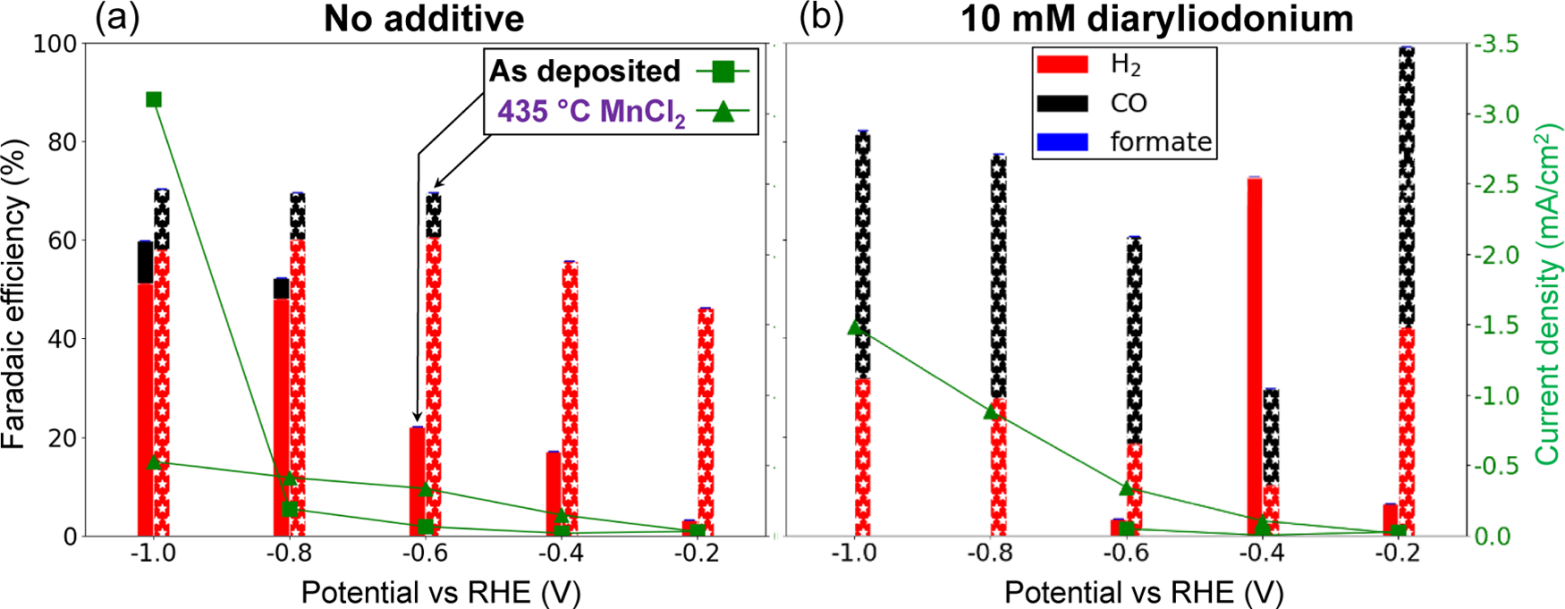
Faradaic efficiency (left axis) and current density (right axis) for the as-deposited (1st bars; squares) and 435 °C MnCl_2_ (2nd bars; triangles) samples without (a) and with 10 mM diaryliodonium additive (b) as a function of potential vs RHE, showing that the MnCl_2_ sample with additive at −1.0 V vs RHE has the best photocurrent density and Faradaic efficiency (−1.5 mA/cm^2^ and 50.4%, respectively).

The diaryliodonium additive increases Faradaic efficiency of CO formation substantially for all sample types (Figures 8 and S12) and makes CO_2_ RR the major reaction in comparison to HER. With this enhanced CO selectivity, the MnCl_2_-treated ZnTe absorber with additive achieves a Faradaic efficiency of 50.4% with a photocurrent density of −1.5 mA/cm^2^ at −1 V vs RHE. For a 2.16 eV MnCl_2_-treated ZnTe absorber (band gap measured in Figure 6), a 1 sun AM1.5G solar resource has 29.8 mW/cm^2^ of above-band gap photons
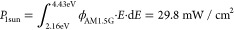
1
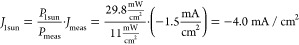
2Here, *P*_1sun_ is the solar resource power density at 1 sun, 2.16 eV is the absorber band gap, 4.43 eV is the maximum energy of the AM1.5G solar irradiance data, φ_AM1.5G_ is the solar irradiance, *E* is the photon energy, *J*_1sun_ is the current density expected at 1 sun, *P*_meas_ is the power density used in the measurement, and *J*_meas_ is the measured current density. Therefore, our −1.5 mA/cm^2^ photocurrent density measured at 11 mW/cm^2^ of 455 nm LED illumination extrapolates to −4.0 mA/cm^2^ at 1 sun AM1.5G. The −4.0 mA/cm^2^ photocurrent would be the best photocurrent density at 1 sun for PEC CO_2_ RR using planar ZnTe compared to prior literature reports (Figure 1 and Table S2). We note that adding Cu, Ag, or Au catalysts may be routes to build on the bulk property improvements of the chloride treatments and further enhance ZnTe absorbers for PEC CO_2_ RR. While we input energy with electrical bias to drive PEC CO_2_ RR in this study, ZnTe’s wide band gap and our transparent substrates leave ample solar resource for adding a PV device in tandem for unassisted operation (i.e., artificial photosynthesis).^55^

## Conclusions

In this work, we introduce chloride treatments as a path to improved ZnTe photoabsorber properties. We sputter ZnTe films and then heat treat them with manganese chloride or magnesium chloride to improve their optoelectronic quality, similar to the CdCl_2_ treatment of CdTe PV absorbers. These chloride treatments are shown to regrow ZnTe crystals for increased grain size, alter the preferential grain orientation, improve the PL intensity, and enhance PEC CO_2_ RR photocurrent density. Additional coupling of the 10 mM diaryliodonium additive to the electrolyte increased the Faradaic efficiency of the improved ZnTe photoelectrodes toward CO formation compared to H_2_ generation. These results open a door to further improvement of ZnTe and related chalcogenide photoabsorber quality for their future use in PEC CO_2_ RR applications.

## Supporting Information Available

Additional materials including XRF data for all samples, XRF data for different H_2_O levels, PL data for new and old chloride sources, XRD data on a logarithmic scale for all samples, UV/visible spectroscopy data for all samples, TRPL data for all samples, quantitative PL and TRPL data for all samples, PEC current density voltage data for all samples, photograph of all samples post-PEC run, CV scans for the bare substrates, ICP–MS data for all samples, PL uniformity data for all samples, Faradaic efficiency and current density data for all samples, and summary of ZnTe PEC CO_2_ RR literature results (PDF)
