# Supplementary material for: Chloride Treatments Improve Zinc Telluride Absorbers for Photoelectrochemical Carbon Dioxide Reduction
Source: ACS Appl Energy Mater. 2025 Jan 7;8(2):983–90. doi: 10.1021/acsaem.4c02498 (PMC11775882; doi:10.1021/acsaem.4c02498)
Supplement: Supplementary file 1 — ae4c02498_si_001.pdf [file ae4c02498_si_001.pdf]

Supporting Information

# Chloride treatments improve zinc telluride absorbers for photoelectrochemical carbon dioxide reduction

Christopher P. Muzzillo,[1,*] Yungchieh Lai,[2] Joel A. Haber,[2] and Andriy Zakutayev[1]

1. National Renewable Energy Laboratory, Golden, Colorado 80401, United States
2. California Institute of Technology, Pasadena, California 91125, United States

* Corresponding author email: christopher.muzzillo@nrel.gov

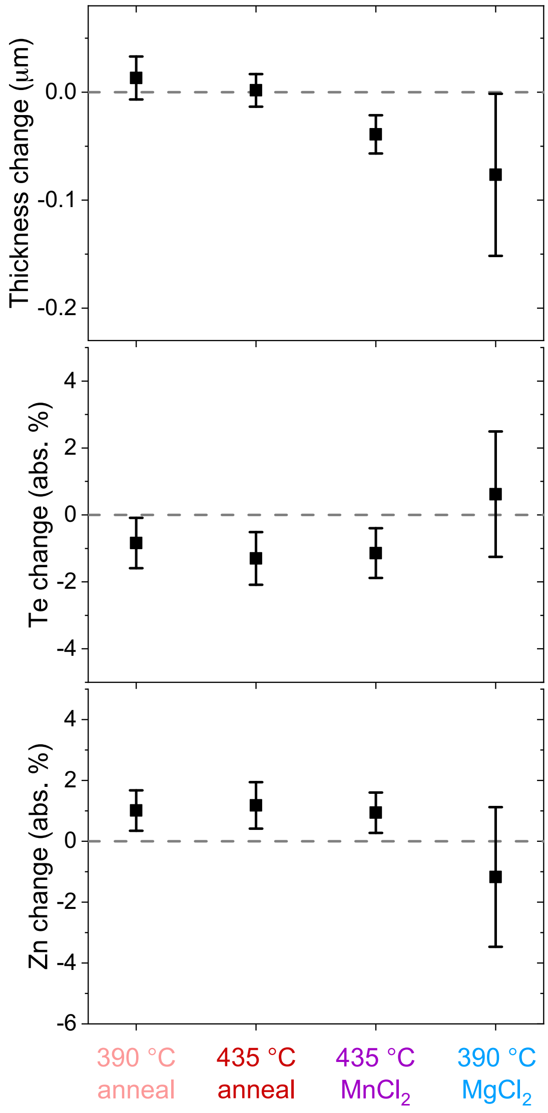


Fig. S1. XRF data showing the change in ZnTe film thickness, Te, and Zn composition after a 390 °C anneal, 435 °C anneal, 435 °C $MnCl_2$ treatment, or 390 °C $MgCl_2$ treatment, showing that anneals cause slight Te loss, while chloride treatments cause more significant Zn loss, etching the film.

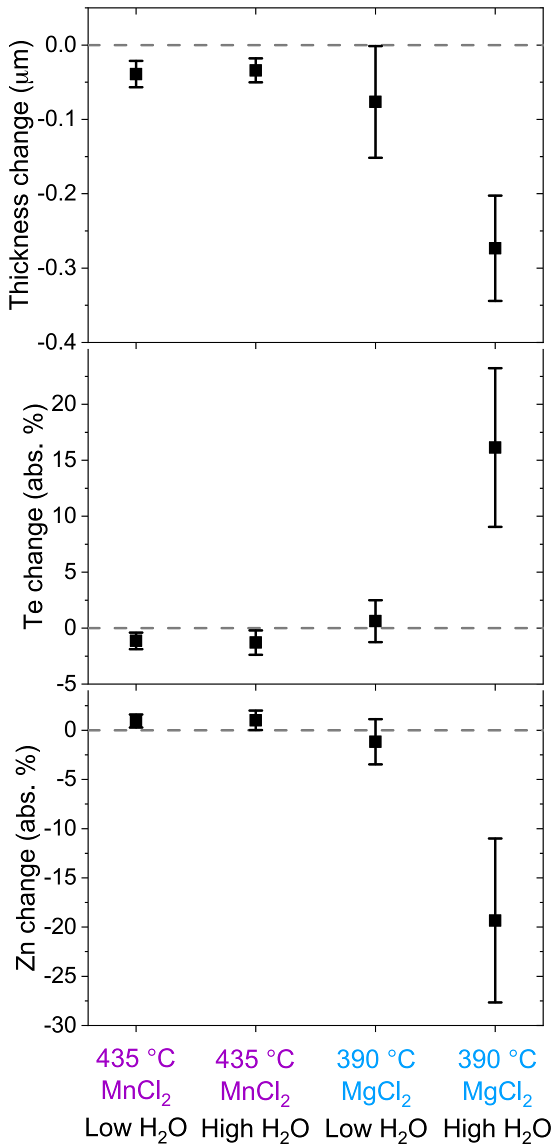


Fig. S2. XRF data showing the change in ZnTe film thickness, Te, and Zn composition after a 435 °C $MnCl_2$ treatment with low or high $H_2O$, or 390 °C $MgCl_2$ treatment with low or high $H_2O$, showing that the 3 $H_2O$ molecules desorbed during the $MnCl_2{\cdot}4H_2O$ pre-bake have a small effect on etching, while the 6 $H_2O$ molecules desorbed during the $MgCl_2{\cdot}6H_2O$ pre-bake have a strong effect on etching.

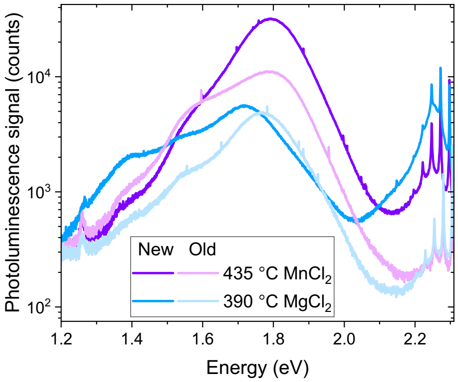


Fig. S3. PL on 435 °C $MnCl_2$ (purple) and 390 °C $MgCl_2$ (blue) samples with new (dark lines) or old (light lines) source material (new and old $MnCl_2$ exposed to high temperature for 4.5 h and 6.5 h, respectively; new and old $MgCl_2$ exposed to high temperature for 1 h and 4.5 h, respectively), showing that the sources become less effective with high temperature exposure, probably due to chlorine loss via HCl formation.

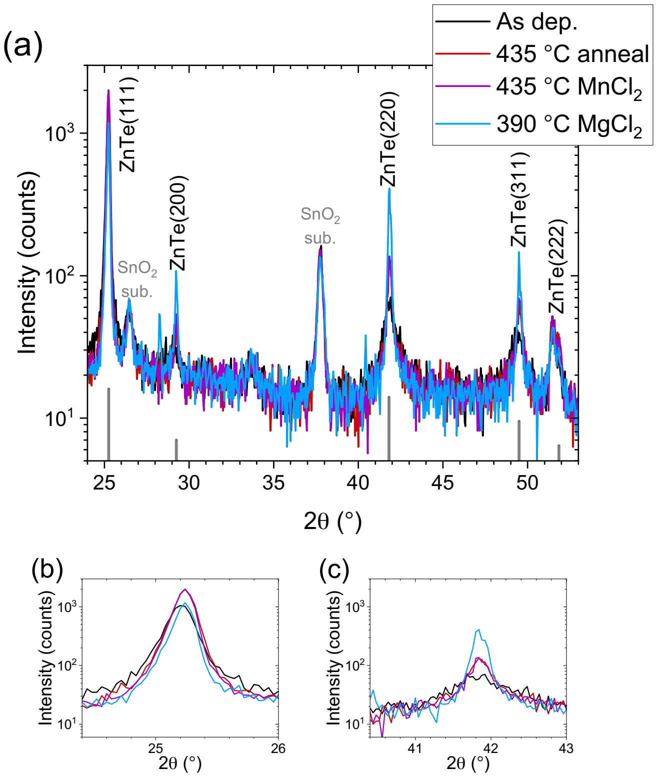


Fig. S4. XRD for the as deposited (black), 435 °C anneal (red), 435 °C $MnCl_2$ (purple), and 390 °C $MgCl_2$ (blue) samples and ICDD powder reference (gray) (a), with closeups of the ZnTe(111) (b) and ZnTe(220) (c), showing that annealing enhances crystallinity and the chloride treatments additionally reduce (111) texture, indicating recrystallization.

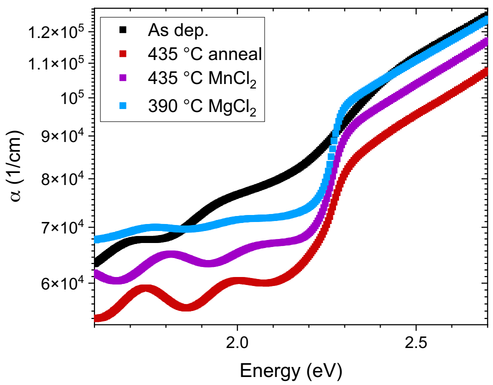


Fig. S5. UV/visible spectroscopy absorption coefficient for the as deposited (black), 435 °C anneal (red), 435 °C $MnCl_2$ (purple), and 390 °C $MgCl_2$ (blue) samples, showing that annealing sharpens the absorption onset and the chloride treatments sharpen it even more.

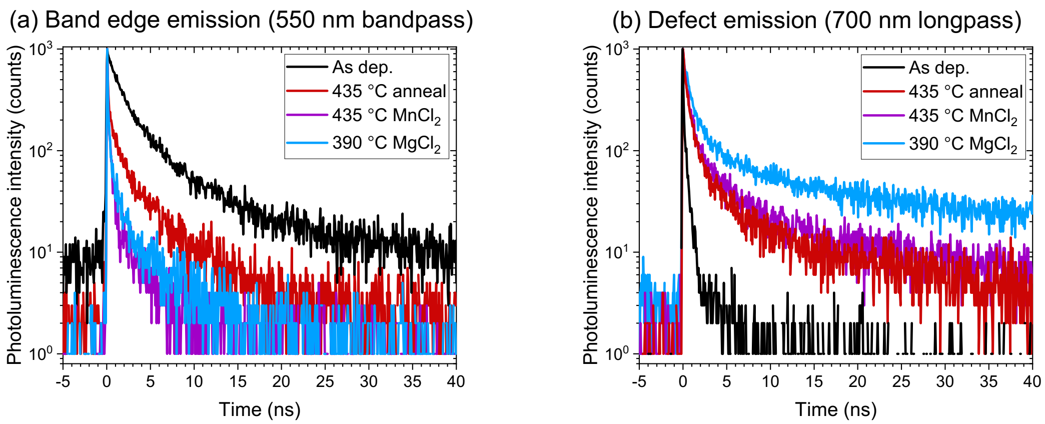


Fig. S6. TRPL for the as deposited (black), 435 °C anneal (red), 435 °C $MnCl_2$ (purple), and 390 °C $MgCl_2$ (blue) samples' band edge emission (a) and defect emission (b), showing that the anneals and chloride treatments reduce band edge TRPL lifetime but enhance defect emission lifetime. More study is needed to put minority carrier lifetimes into context with data on carrier concentration and mobility.

Table S1. Band edge (550 nm) and defect (700 nm) PL mean intensity with standard deviation from measurements at multiple locations, short TRPL lifetime ($\tau_1$), and long TRPL lifetime ($\tau_2$) for the samples, showing that the chloride treatments improve band edge luminescent quantum yield but more study is needed to understand their effect on lifetimes.

| Sample | Band edge (550 nm) | | | Defect (700 nm) | | |
|---|---|---|---|---|---|---|
| | PL intensity (counts) | TRPL $\tau_1$ (ns) | TRPL $\tau_2$ (ns) | PL intensity (counts) | TRPL $\tau_1$ (ns) | TRPL $\tau_2$ (ns) |
| As dep. | 152 ± 14 | 1.59 | 11.3 | 199 ± 5 | 0.10 | 0.62 |
| 435 °C anneal | 788 ± 48 | 0.09 | 2.43 | 39,490 ± 2,210 | 0.43 | 4.95 |
| 435 °C $MnCl_2$ | 3,918 ± 3,475 | 0.07 | 0.60 | 27,230 ± 5,960 | 0.52 | 8.87 |
| 390 °C $MgCl_2$ | 21,830 ± 12,280 | 0.07 | 1.31 | 9,411 ± 2,450 | 0.87 | 25.1 |

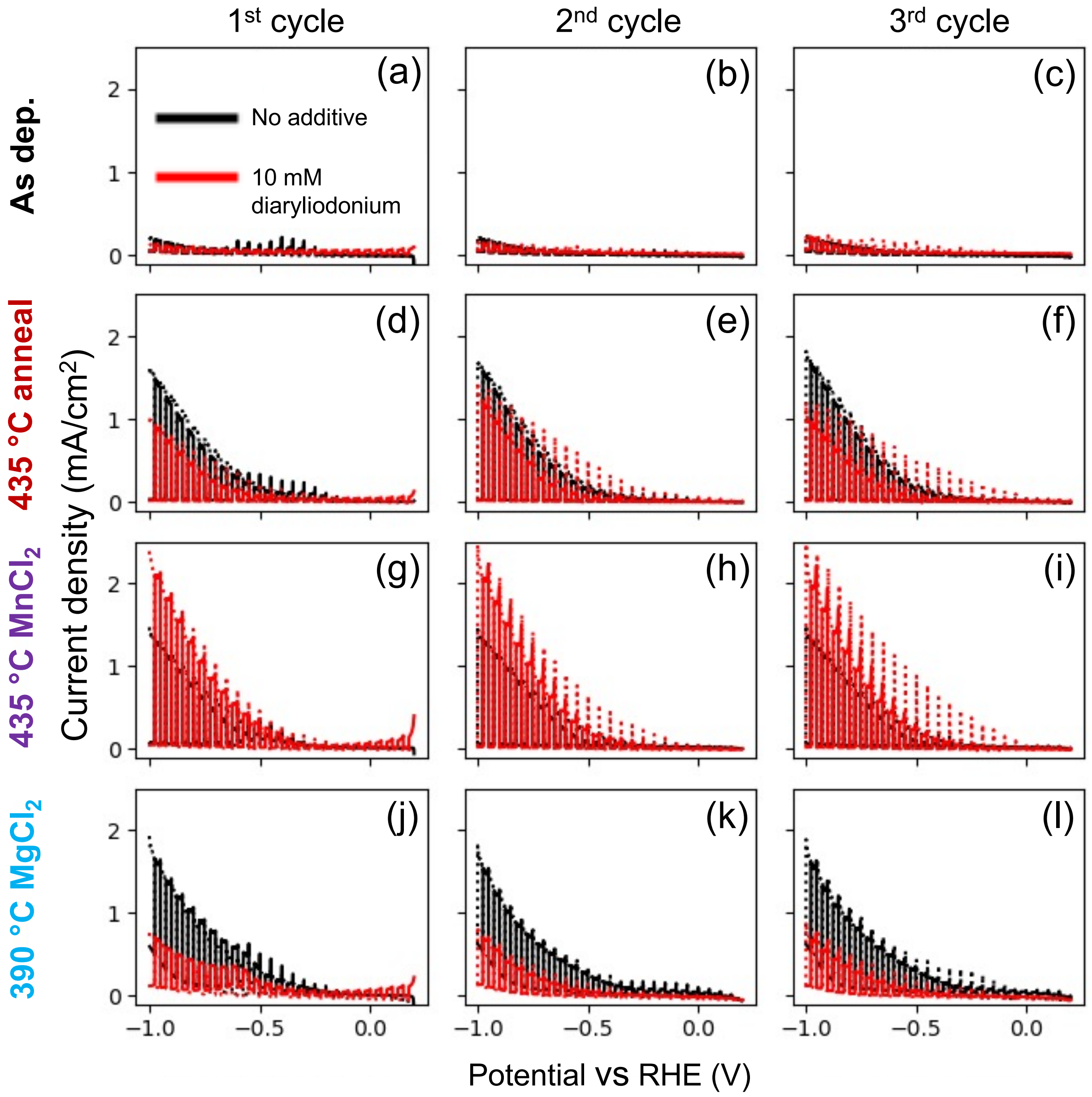


Fig. S7. PEC current density-voltage data without (black lines) and with 10 mM diaryliodonium additive (red lines) for the as deposited (a) – (c), 435 °C anneal (d) – (f), 435 °C $MnCl_2$ (g) – (i), and 390 °C $MgCl_2$ (j) – (l) samples, respectively. The solid and dotted lines are cathodic and anodic sweeps, respectively. The 1st cycle is in (a), (d), (g), and (j), the 2nd cycle is in (b), (e), (h), and (k), and the 3rd cycle is in (c), (f), (i), and (l) for each sample, showing that the $MnCl_2$ sample with additive has the greatest photocurrent density: -1.5 mA/cm$^2$ for at -1.0 V vs RHE and 11 mW/cm$^2$ illumination with a 455 nm LED.

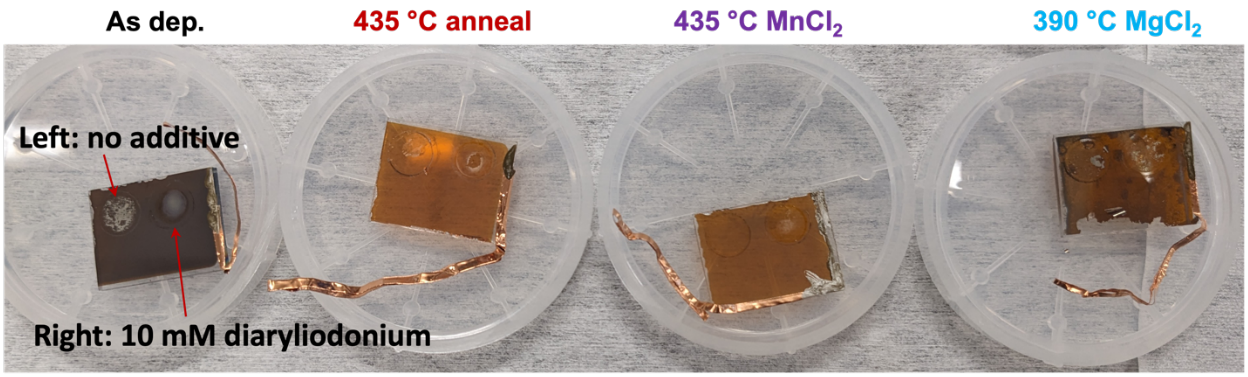


Fig. S8. Picture of post-PEC run ZnTe samples.

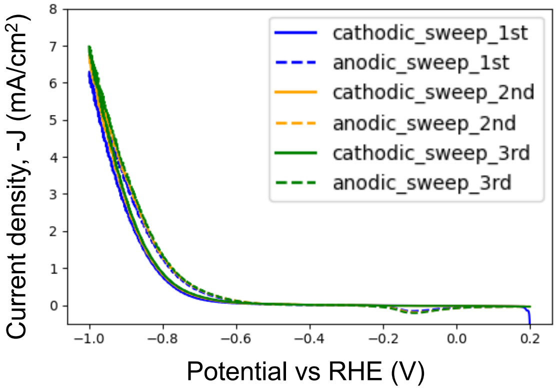


Fig. S9. CV scans with light chopping for the bare F:$SnO_2$ substrate. The test was operated with 0.1 M $KHCO_3$ electrolyte without additives, 455 nm light-emitting diode illumination at 11 mW/cm$^2$, and 10 mV/s sweeps from +0.2 to -1.0 V vs reversible hydrogen electrode (RHE). This substrate shows only dark current at potential more negative than -0.7 V vs RHE and not any photocurrent.

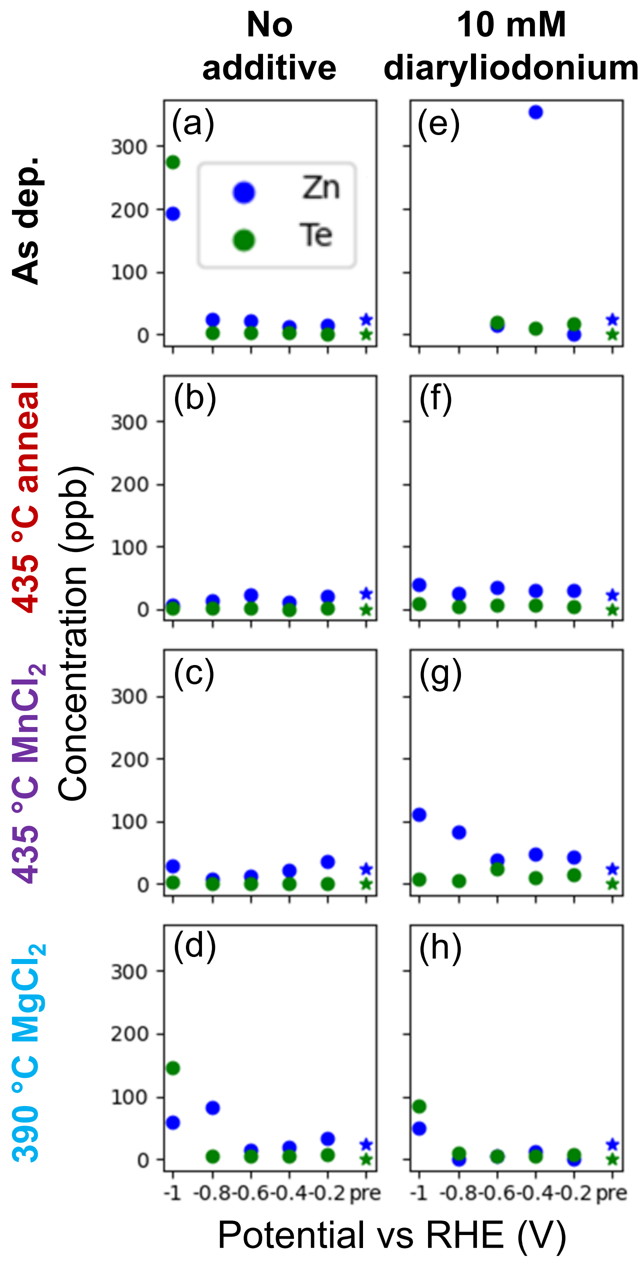


Fig. S10. ICP-MS results for post-PEC run electrolytes (circles), showing very little ZnTe dissolution compared to the pre-PEC electrolyte (star symbols) for the as deposited (a) and (e), 435 °C anneal (b) and (f), 435 °C $MnCl_2$ (c) and (g), and 390 °C $MgCl_2$ (d) and (h) samples without and with additive, respectively, indicating that the ZnTe is stable for most sample types and conditions tested.

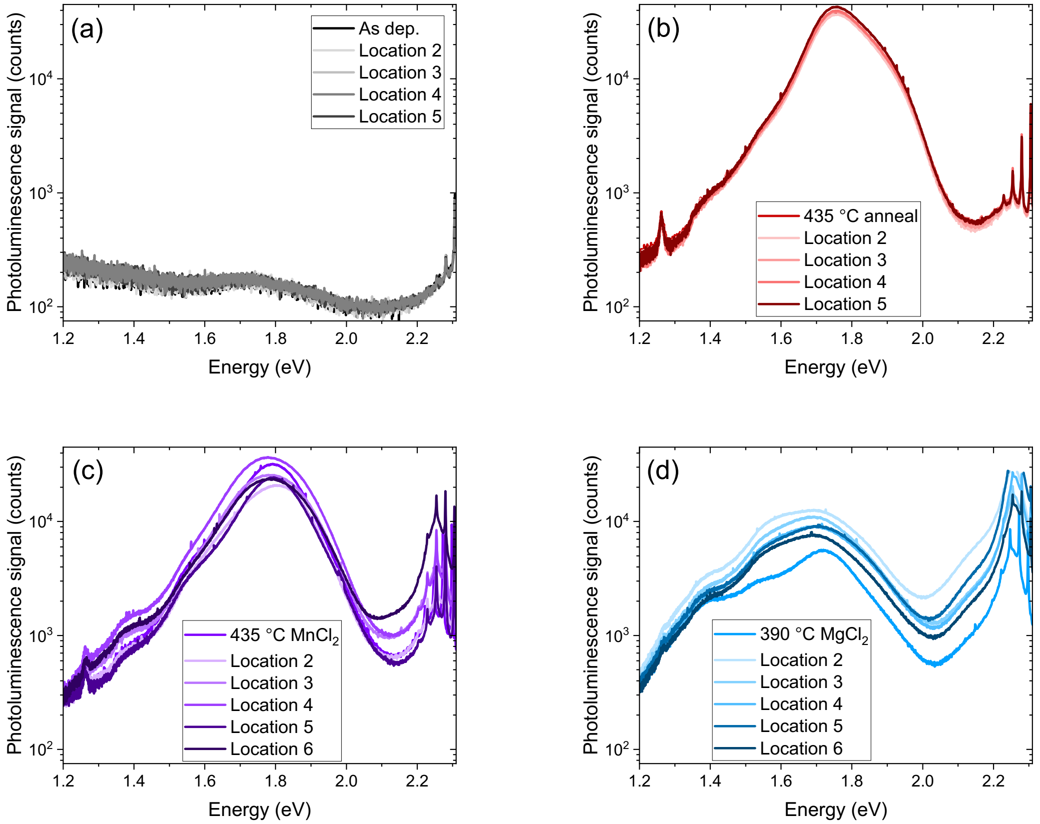


Fig. S11. PL uniformity for the as deposited (a), 435 °C anneal (b), 435 °C $MnCl_2$ (c), and 390 °C $MgCl_2$ (d) samples, showing that the chloride treatments introduce nonuniformity in optoelectronic quality, where more uniform source material may eliminate this issue in the future.

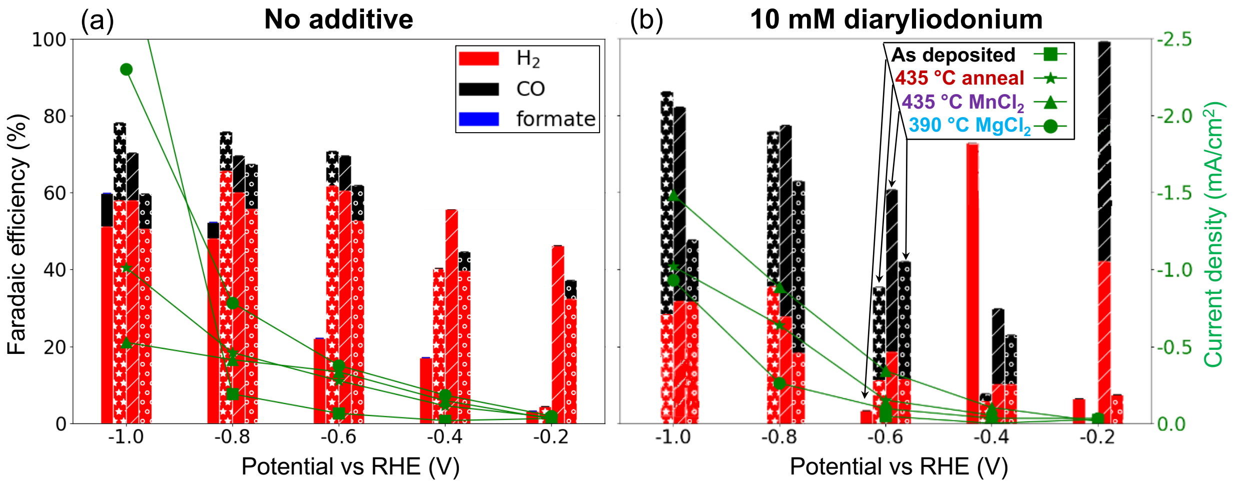


Fig. S12. Faradaic efficiency (left axis) and current density (right axis) for the as deposited (1st bars; squares), 435 °C anneal (2nd bars; stars), 435 °C $MnCl_2$ (3rd bars; triangles), and

390 °C $MgCl_2$ (4th bars; circles) samples without (a) and with 10 mM diaryliodonium additive (b) as a function of potential vs RHE, showing that the additive enhances the faradaic efficiency of CO formation for all sample types and the $MnCl_2$ sample with additive at -1.0 V vs RHE has the best photocurrent density and faradaic efficiency (-1.5 mA/cm$^2$ and 50.4%, respectively).

Table S2. Literature results for ZnTe PEC $CO_2$ RR: ZnTe device structure (planar or nanostructured) and conditions: Potential vs RHE, $CO_2$ RR overpotential (-1.57 V vs $Fc^+/Fc$ in acetonitrile[1]), photocurrent density, faradaic efficiency (FE), product of negative photocurrent density and faradaic efficiency (-J*FE), $CO_2$ RR product, electrolyte, illumination, and reference.

| ZnTe device structure | V vs RHE (V) | Overpot. (mV) | Photocurr. dens. (mA/cm$^2$) | FE (%) | -J*FE | Product | Electrolyte | Illumination | Reference |
|---|---|---|---|---|---|---|---|---|---|
| Planar | -1 | 0.89 | -1.5 | 50.4 | 75.6 | CO | 0.1 M $KHCO_3$ | 11 mW/cm$^2$ 455 nm LED | This work |
| | -1 | 0.89 | -4 | 50.4 | 201.6 | CO | 0.1 M $KHCO_3$ | Extrap. to 1 sun | This work |
| | - | 1.01 | -2.1 | 69 | 144.9 | CO | Acetonitrile | 1 sun | [2] |
| | -0.7 | 0.59 | -2.76 | 39.7 | 109.6 | CO | 0.1 M $KHCO_3$ | 1 sun | [3] |
| | - | 0.81 | -0.43 | 29.4 | 12.6 | CO | Acetonitrile | 1 sun | [4] |
| | -0.3 | 0.19 | -0.03 | 26.1 | 0.8 | HCOOH | 0.1 M $KHCO_3$ | 1 sun(?) | [5] |
| | -1 | 0.89 | -0.1 | 3.4 | 0.3 | CO | 0.5 M $KHCO_3$ | 1 sun | [6] |
| Nano | -0.7 | 0.59 | -16 | 58 | 928.0 | CO | 0.5 M $KHCO_3$ | 1 sun | [6] |
| | - | 1.01 | -7.7 | 81 | 623.7 | CO | Acetonitrile | 1 sun | [2] |
| | - | 0.91 | -6.67 | 92.9 | 619.6 | CO | Acetonitrile | 1 sun | [4] |
| | -0.5 | 0.39 | -9.2 | 64.6 | 594.3 | CO | 0.1 M $KHCO_3$ | 1 sun | [3] |
| | -0.11 | 0 | -3.88 | 65.82 | 255.4 | CO | 0.5 M $KHCO_3$ | 1 sun | [7] |
| | - | 0.21 | -2.35 | 87 | 204.5 | CO | Acetonitrile | 1 sun | [8] |
| | -0.7 | 0.59 | -8.2 | 22.9 | 187.8 | CO | 0.5 M $KHCO_3$ | 1 sun | [9] |
| | -0.11 | 0 | -1.21 | 71.65 | 86.7 | CO | 0.5 M $KHCO_3$ | 1 sun | [10] |
| | -0.11 | 0 | -1.1 | 60 | 66.0 | CO | 0.5 M $KHCO_3$ | 1 sun | [11] |
| | -0.59 | 0.48 | -0.26 | 79 | 20.6 | $C_2H_5OH$ | 0.1 M $KHCO_3$ | 1 sun | [12] |

## References


(1) Matsubara, Y. Standard Electrode Potentials for the Reduction of $CO_2$ to CO in Acetonitrile–Water Mixtures Determined Using a Generalized Method for Proton-Coupled Electron-Transfer Reactions. *ACS Energy Lett.* **2017,** *2* (8), 1886-1891, DOI: 10.1021/acsenergylett.7b00548.
(2) Wei, Y.; Zhu, Y.; Li, P.; Gao, X.; Yu, Z.; Liu, S.; Li, N.; Shen, Y.; Wang, M. Surface states modulation of ZnTe via ultrathin ZnO layer as efficient photocathodes for $CO_2$ reduction reaction. *Appl. Catal. B-Environ.* **2024,** *347*, 123760, DOI: 10.1016/j.apcatb.2024.123760.
(3) Wen, P.; Li, H.; Ma, X.; Lei, R.; Wang, X.; Geyer, S. M.; Qiu, Y. A colloidal ZnTe quantum dot-based photocathode with a metal–insulator–semiconductor structure towards solar-

driven $CO_2$ reduction to tunable syngas. *J. Mater. Chem. A* **2021,** *9* (6), 3589-3596, DOI: 10.1039/D0TA10394B.
(4) Wang, Q.; Gao, X.; Wei, Y.; Liu, T.; Huang, Q.; Ren, D.; Zakeeruddin, S. M.; Grätzel, M.; Wang, M.; Li, Q.; Yang, J.; Shen, Y. Boosting Interfacial Electron Transfer and $CO_2$ Enrichment on ZIF-8/ZnTe for Selective Photoelectrochemical Reduction of $CO_2$ to CO. *ACS Appl. Mater. Interfaces* **2024,** *16* (28), 36462-36470, DOI: 10.1021/acsami.4c06921.
(5) Won, D. H.; Chung, J.; Park, S. H.; Kim, E.-H.; Woo, S. I. Photoelectrochemical production of useful fuels from carbon dioxide on a polypyrrole-coated p-ZnTe photocathode under visible light irradiation. *J. Mater. Chem. A* **2015,** *3* (3), 1089-1095, DOI: 10.1039/C4TA05901H.
(6) Jang, Y. J.; Jang, J.-W.; Lee, J.; Kim, J. H.; Kumagai, H.; Lee, J.; Minegishi, T.; Kubota, J.; Domen, K.; Lee, J. S. Selective CO production by Au coupled ZnTe/ZnO in the photoelectrochemical $CO_2$ reduction system. *Energy Environ. Sci.* **2015,** *8* (12), 3597-3604, DOI: 10.1039/C5EE01445J.
(7) Jang, Y. J.; Jeong, I.; Lee, J.; Lee, J.; Ko, M. J.; Lee, J. S. Unbiased Sunlight-Driven Artificial Photosynthesis of Carbon Monoxide from $CO_2$ Using a ZnTe-Based Photocathode and a Perovskite Solar Cell in Tandem. *ACS Nano* **2016,** *10* (7), 6980-6987, DOI: 10.1021/acsnano.6b02965.
(8) Gao, X.; Li, N.; Li, P.; Wei, Y.; Huang, Q.; Akhtar, K.; Bakhsh, E. M.; Khan, S. B.; Shen, Y.; Wang, M. ZnTe/$SnS_2$ heterojunction for photo-electrocatalysis of $CO_2$ to CO. *Electrochim. Acta* **2024,** *497*, 144603, DOI: 10.1016/j.electacta.2024.144603.
(9) Jang, J.-W.; Cho, S.; Magesh, G.; Jang, Y. J.; Kim, J. Y.; Kim, W. Y.; Seo, J. K.; Kim, S.; Lee, K.-H.; Lee, J. S. Aqueous-Solution Route to Zinc Telluride Films for Application to $CO_2$ Reduction. *Angew. Chem. Int. Ed.* **2014,** *53* (23), 5852-5857, DOI: 10.1002/anie.201310461.
(10) Jang, Y. J.; Bhatt, M. D.; Lee, J.; Choi, S. H.; Lee, B. J.; Lee, J. S. Metal-Free Artificial Photosynthesis of Carbon Monoxide Using N-Doped ZnTe Nanorod Photocathode Decorated with N-Doped Carbon Electrocatalyst Layer. *Adv. Energy Mater.* **2018,** *8* (20), 1702636, DOI: 10.1002/aenm.201702636.
(11) Jang, Y. J.; Lee, C.; Moon, Y. H.; Choe, S. Solar-Driven Syngas Production Using Al-Doped ZnTe Nanorod Photocathodes. *Materials* **2022,** *15* (9), 3102, DOI: 10.3390/ma15093102.
(12) Wang, Q.; Wang, X.; Yu, Z.; Jiang, X.; Chen, J.; Tao, L.; Wang, M.; Shen, Y. Artificial photosynthesis of ethanol using type-II g-$C_3N_4$/ZnTe heterojunction in photoelectrochemical $CO_2$ reduction system. *Nano Energy* **2019,** *60*, 827-835, DOI: 10.1016/j.nanoen.2019.04.037.
